# Neutrophil extracellular traps are associated with disease severity and microbiota diversity in patients with chronic obstructive pulmonary disease

**DOI:** 10.1016/j.jaci.2017.04.022

**Published:** 2018-01

**Authors:** Alison J. Dicker, Megan L. Crichton, Eleanor G. Pumphrey, Andrew J. Cassidy, Guillermo Suarez-Cuartin, Oriol Sibila, Elizabeth Furrie, Christopher J. Fong, Wasyla Ibrahim, Gill Brady, Gisli G. Einarsson, J. Stuart Elborn, Stuart Schembri, Sara E. Marshall, Colin N.A. Palmer, James D. Chalmers

**Affiliations:** a Scottish Centre for Respiratory Research, University of Dundee, Ninewells Hospital and Medical School, Dundee, United Kingdom; b Respiratory Department, Hospital de la Santa Creu i Sant Pau, Institut d'Invesitgacio Biomedica (IIB) Sant Pau, Barcelona, Spain; c Centre for Infection and Immunity, School of Medicine, Dentistry and Biomedical Sciences, Queen's University Belfast, Belfast, United Kingdom; d National Heart and Lung Institute, Imperial College London, London, United Kingdom; e Division of Molecular & Clinical Medicine, School of Medicine, University of Dundee, and the Wellcome Trust, London, United Kingdom

**Keywords:** Neutrophils, phagocytosis, chronic obstructive pulmonary disease, *Haemophilus* species, exacerbations, CAT, COPD Assessment Test, cfDNA, Cell-free DNA, COPD, Chronic obstructive pulmonary disease, EN-RAGE, Ligand for the receptor for advanced glycation end products, GOLD, Global Initiative for Obstructive Lung Disease, ICS, Inhaled corticosteroid, MPO, Myeloperoxidase, MRC, Medical Research Council, NET, Neutrophil extracellular trap, OTU, Operational taxonomic unit, PMA, Phorbol 12-myristate 13-acetate, SWDI, Shannon-Wiener species diversity index

## Abstract

**Background:**

Neutrophil extracellular traps (NETs) have been observed in the airway in patients with chronic obstructive pulmonary disease (COPD), but their clinical and pathophysiologic implications have not been defined.

**Objective:**

We sought to determine whether NETs are associated with disease severity in patients with COPD and how they are associated with microbiota composition and airway neutrophil function.

**Methods:**

NET protein complexes (DNA-elastase and histone-elastase complexes), cell-free DNA, and neutrophil biomarkers were quantified in soluble sputum and serum from patients with COPD during periods of disease stability and during exacerbations and compared with clinical measures of disease severity and the sputum microbiome. Peripheral blood and airway neutrophil function were evaluated by means of flow cytometry *ex vivo* and experimentally after stimulation of NET formation.

**Results:**

Sputum NET complexes were associated with the severity of COPD evaluated by using the composite Global Initiative for Obstructive Lung Disease scale (*P* < .0001). This relationship was due to modest correlations between NET complexes and FEV_1_, symptoms evaluated by using the COPD assessment test, and higher levels of NET complexes in patients with frequent exacerbations (*P* = .002). Microbiota composition was heterogeneous, but there was a correlation between NET complexes and both microbiota diversity (*P* = .009) and dominance of *Haemophilus* species operational taxonomic units (*P* = .01). *Ex vivo* airway neutrophil phagocytosis of bacteria was reduced in patients with increased sputum NET complexes. Consistent results were observed regardless of the method of quantifying sputum NETs. Failure of phagocytosis could be induced experimentally by incubating healthy control neutrophils with soluble sputum from patients with COPD.

**Conclusion:**

NET formation is increased in patients with severe COPD and associated with more frequent exacerbations and a loss of microbiota diversity.

## Graphical abstract

**Figure:**
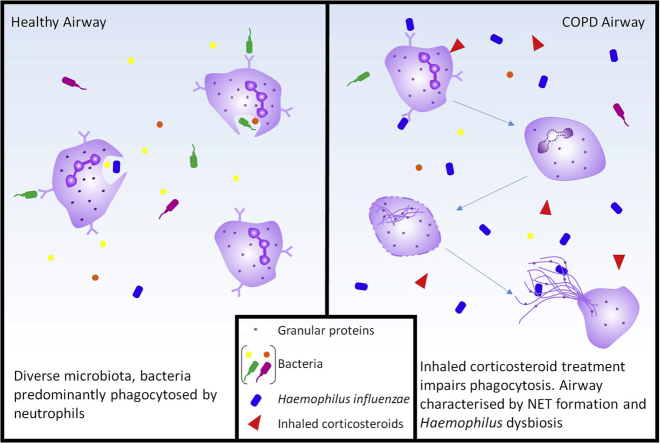

Chronic obstructive pulmonary disease (COPD) is a heterogeneous disorder caused primarily by cigarette smoking and with multiple phenotypes and an unpredictable clinical course; drivers of disease progression remain poorly understood.^1^, ^2^, ^3^, ^4^ Aberrant neutrophilic inflammation is characteristic of COPD, and neutrophils contribute to airway damage through release of proteases and reactive oxygen species,^5^ leading to loss of alveoli, increased mucus production, and mucociliary dysfunction. Normally, activated neutrophils rapidly undergo apoptosis and are removed by alveolar macrophages in a noninflammatory manner. This process is essential in resolving inflammation and preventing disease progression, and therefore neutrophil phagocytosis is a crucial defense against bacterial infection but also important in resolving inflammation and limiting disease progression in patients with COPD.^6^, ^7^, ^8^ Cigarette smoke directly promotes neutrophilic inflammation but also impairs this antibacterial defense, leading to disturbance of the resident microbiota, which in turn promotes neutrophil influx and exacerbates inflammation.^9^, ^10^

An alternate method of neutrophil antimicrobial defense, called neutrophil extracellular trap (NET) formation or NETosis, has been described.^11^ This is an extracellular method of pathogen trapping in which neutrophils extrude webs of decondensed chromatin studded with histones, neutrophil elastase, and other granule products that ensnare bacteria. Although the ability of NETs to ensnare target microorganisms is not in doubt, their direct role in bacterial killing remains controversial.^11^, ^12^ The cellular mechanisms that mediate lytic NET formation are still to be elucidated, but evidence is accumulating that neutrophil elastase plays a central role, initially translocating from cytoplasmic granules to the nucleus, where it instigates chromatin degradation through histone cleavage.^13^

Recently, NETs have been identified in the sputum of small numbers of patients with stable and exacerbating COPD through the use of confocal fluorescent and electron microscopy.^14^, ^15^, ^16^ In the study by Grabcanovic-Musija et al,^14^ COPD disease severity, as measured based on lung function, was associated with a greater amount of NET-associated neutrophil elastase determined by using confocal laser microscopy. However, the clinical and pathophysiologic relevance of NETs in patients with COPD has not been established. In this study we used multiple methods to evaluate airway NET release and correlated them with clinical disease severity, the airway microbiome, and neutrophil function. We demonstrate that NETs are more abundant in patients with severe COPD and are associated with more frequent exacerbations, reduced microbiota diversity, and an abundance of *Haemophilus* species.

## Methods

Patients with COPD enrolled in a community COPD registry (the Tayside Allergy and Respiratory Disease Information System)^17^, ^18^ were recruited into this prospective longitudinal cohort study. Patients were included if they were older than 40 years, had an FEV_1_/forced vital capacity ratio of less than 70%, and had a clinical diagnosis of COPD. Exclusion criteria included the inability to provide informed consent, previous adverse reaction to nebulized hypertonic saline, asthma, bronchiectasis on high-resolution computed tomographic scanning, cystic fibrosis, active mycobacterial disease, and immunosuppression. Patients receiving long-term antibiotic therapy or maintenance oral corticosteroid therapy at screening were also excluded. Study approval was granted by the East of Scotland Research Ethics Committee (13/ES/0030), and all patients provided written informed consent to participate.

### Study design

Patients underwent a comprehensive clinical assessment and sampling of blood and sputum at 2 time points up to 6 months apart while clinically stable. Exacerbations were reported to the research team, who provided standardized treatment with repeat clinical assessment and blood and sputum sampling at the onset of exacerbation and at day 10 after treatment. Exacerbations were defined, as previously described.^19^ Relevant medical history was recorded at screening (see the Methods section in this article's Online Repository at www.jacionline.org for details). Sputum was obtained after nebulization of 3% hypertonic saline for up to 20 minutes. Spirometry was performed, and the St Georges Respiratory Questionnaire, COPD Assessment Test (CAT) and Medical Research Council (MRC) Dyspnoea Score were used at each visit. The primary outcome was the association between NET complexes and composite Global Initiative for Obstructive Lung Disease (GOLD) COPD severity classification, which classifies patients into 4 groups, A, B, C, and D, depending on their symptoms (CAT score and MRC Dyspnoea score), lung function (FEV_1_ percent predicted), and exacerbation frequency (high risk defined as ≥2 exacerbations per year or hospitalization for a severe exacerbation).^20^

### NET assays

There is no agreed upon high-throughput method of quantifying NETs in biological fluids, and consequently, this study used multiple methods. First, primary NET constituents, including cell-free DNA (cfDNA), myeloperoxidase (MPO), neutrophil elastase, and the ligand for the receptor for advanced glycation end products (EN-RAGE), were quantified.^21^ These assays are not specific because these components are also released during neutrophil degranulation or necrosis but are commonly used as surrogates of NET release.

**Fig E1:**
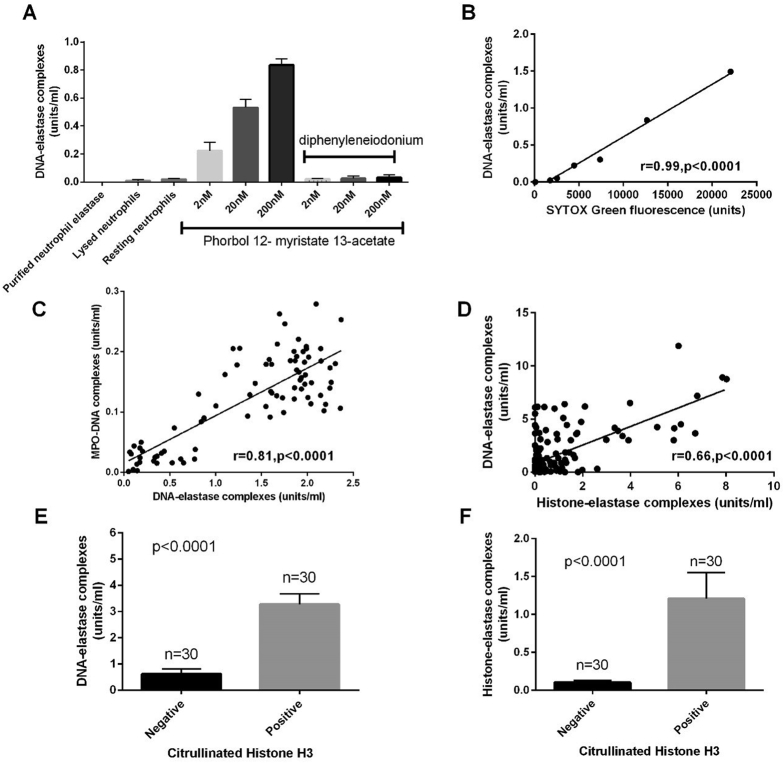


**Fig E2:**
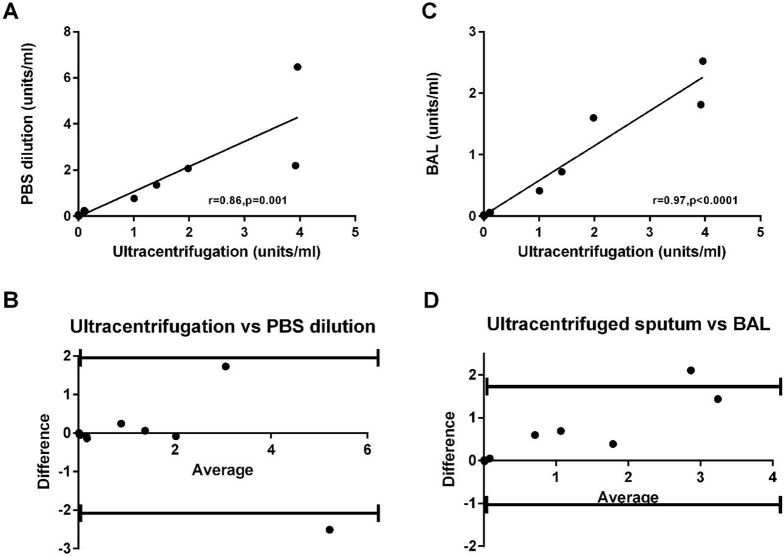


**Fig E3:**
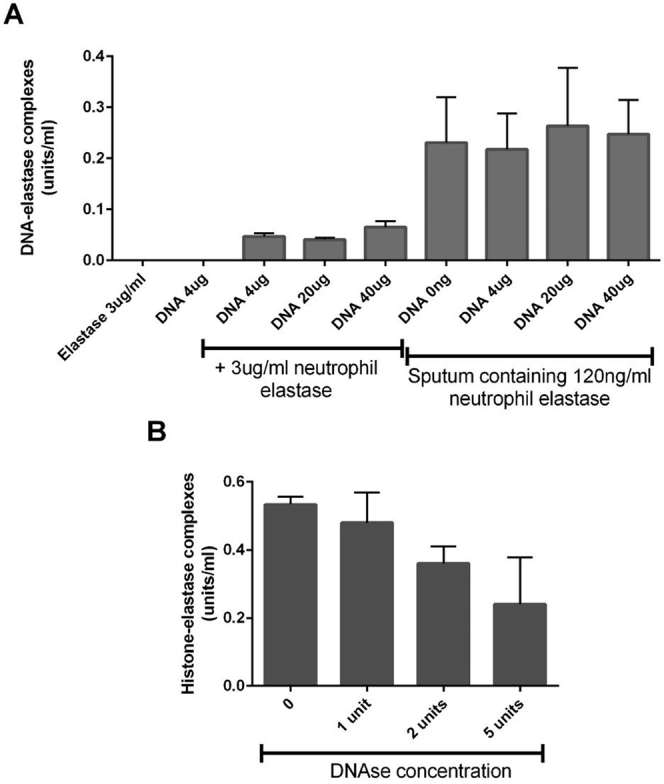


Subsequently, 3 specific methods of NET quantification were used: an MPO-DNA ELISA that has been extensively described in the literature^21^, ^22^, ^23^ and 2 assays developed and validated in house for use in sputum based on detection of DNA-elastase and histone-elastase complexes. For the DNA-elastase complex assay, anti-DNA (HYB331-01; Abcam, Cambridge, United Kingdom) capture antibody was incubated on plates overnight at 4°C, followed by washing with PBS plus 0.05% Tween 20 (wash buffer). Plates were blocked with 1% BSA in PBS and washed with wash buffer. Samples were diluted in 1% BSA in PBS. A standard curve was generated by titrating concentrations of heathy human blood-derived neutrophils treated with phorbol 12-myristate 13-acetate (PMA). Plates were washed 3 times with wash buffer after incubation of standards and samples. DNA-elastase complexes were detected with sheep anti–neutrophil elastase–horseradish peroxidase (PA1-74133; Thermo Scientific, Waltham, Mass) and developed with 3,3′5,5′-tetramethylbenzidine. For the histone-elastase assay, plates were coated for 1 hour with anti–histone H1 (ab71594, Abcam), washed and blocked as above, and incubated for 1 hour with rabbit anti–neutrophil elastase (ab21595, Abcam). Anti-rabbit–horseradish peroxidase (ab6721, Abcam) was used for detection, and the plate was developed as above. The assays were validated against other known NET components (citrullinated histone H3 and DNA) for the effects of sample preparation methods and for passive interactions between DNA and elastase (see Figs E1-E3 in this article's Online Repository at www.jacionline.org).

### Sputum microbiome

DNA was extracted from whole sputum by using the AllPrep DNA/RNA Mini Kit on the QIAcube automation platform (Qiagen, Hilden, Germany) with a modified protocol, followed by 16S rRNA gene sequencing on the Illumina MiSeq platform (Illumina, San Diego, Calif). Bioinformatic analysis and quality checking of the resulting sequences were performed with Quantitative Insights in Microbial Ecology (version 1.9.0).^24^ The Shannon-Wiener Species Diversity Index (SWDI) was used as a measure of α-diversity of samples. See the Methods section in this article's Online Repository for full details.

### Neutrophil studies

Peripheral blood neutrophils were isolated by means of Percoll gradient density centrifugation, as previously described.^6^ Phagocytosis by peripheral blood and airway neutrophils was assessed with a flow cytometry–based assay (see the Methods section in this article's Online Repository for more details).^25^ Sputum neutrophil platelet aggregates were investigated by means of flow cytometry, whereas cytospin preparations were obtained for differential sputum cell counts (see the Methods section in this article's Online Repository for more details).

### Statistical analysis

Details of all statistical analyses carried out are shown in the Methods section in this article's Online Repository.

## Results

Ninety-nine patients were included in the study. Patients' characteristics are shown in Table I.

**Table I:** Demographic and clinical characteristics of the cohort at study entry

	Cohort
Demographics and major comorbidities	
No.	99
Age (SD)	71.3 (8.3)
Age at diagnosis (y [SD])	59.8 (11.5)
Male sex, no. (%)	66 (66.7)
Active smokers, no. (%)	24 (24.2)
Ex-smokers, no. (%)	71 (71.7)
Pack years (SD)	42 (29)
BMI (kg/m^2^ [SD])	28.3 (5.7)
Myocardial infarction, no. (%)	15 (15.2)
CABG, no. (%)	14 (14.1)
Angina, no. (%)	24 (24.2)
Stroke, no. (%)	9 (9.1)
Diabetes, no. (%)	19 (19.2)
Cancer, no. (%)	3 (3.0)
CCF, no. (%)	5 (5.1)
Lung surgery, no. (%)	4 (4.0)
Kidney disease, no. (%)	1 (1.0)
COPD severity	
FEV_1_ (% predicted [SD])	70.3 (21.7)
MRC Dyspnoea Score (SD)	2.8 (1.4)
Exacerbations per year (SD)	2.1 (2.0)
GOLD score, no. (%)	
A	7 (7.1)
B	36 (36.4)
C	5 (5.1)
D	51 (51.5)
SGRQ (SD)	44.5 (22.1)
On LTOT, no. (%)	5 (5.1)
Medications	
Statins, no. (%)	54 (54.6)
ICS, no. (%)	61 (61.6)
LABA, no. (%)	13 (13.1)
LAMA, no. (%)	53 (53.5)
Theophylline, no. (%)	8 (8.1)
Mucolytic, no. (%)	13 (13.1)
Aspirin, no. (%)	26 (26.3)
β-Blocker, no. (%)	13 (13.1)
ACE inhibitor, no. (%)	27 (27.3)
ARB, no. (%)	6 (6.1)
Clopidogrel, no. (%)	8 (8.1)

*ACE*, Angiotensin-converting enzyme; *ARB*, angiotensin receptor blocker; *BMI*, body mass index; *CABG*, coronary artery bypass graft; *CCF*, congestive cardiac failure; *LABA*, long-acting β-agonist; *LAMA*, long-acting muscarinic antagonist; *LTOT*, long-term oxygen therapy; *SGRQ*, St Georges Respiratory Questionnaire.

### NETs are associated with clinical disease severity in patients with stable COPD

**Fig 1:**
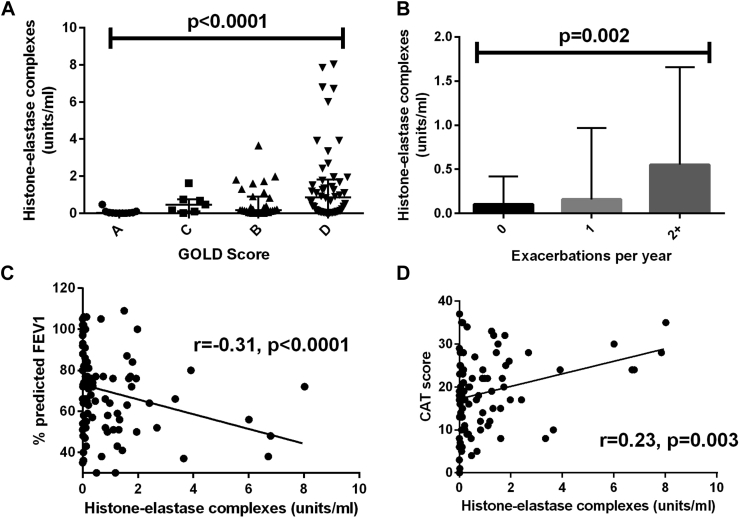
Histone-elastase complex concentrations in soluble sputum of patients with COPD are associated with clinical markers of COPD disease severity. **A,** NET concentration in stable samples compared with GOLD score (n = 99). **B,** NET concentration compared with number of exacerbations reported by study patients in previous year (n = 99). **C,** NET concentration in stable samples compared with percent predicted FEV_1_ (n = 99). **D,** NET concentration in stable samples compared with CAT score (n = 99).

**Fig E4:**
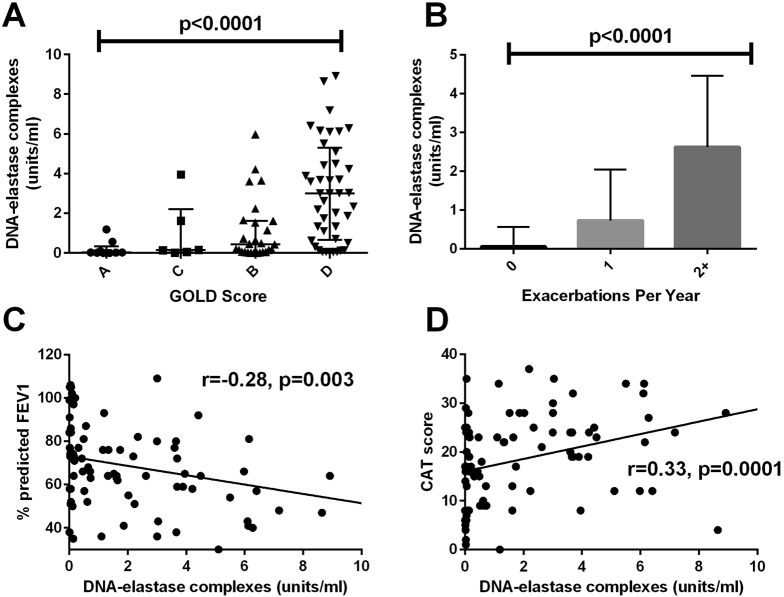


Sputum NET concentrations were measured in expectorated sputum from all subjects. NET concentrations quantified by using the histone-elastase complex assay were associated with multiple markers of COPD severity. Sputum NET concentrations were highest in those in 2011 GOLD group B and D, the most severe groups using this composite index of COPD severity (consisting of lung function [percent predicted FEV_1_], symptoms [MRC Dyspnoea Score and CAT score], and exacerbation frequency; *P* < .0001; Fig 1, *A*).^26^ We explored the individual contributors to the GOLD classification and found that sputum NET concentrations were also correlated independently with annual exacerbation frequency (*P* = .002; Fig 1, *B*), percent predicted FEV_1_ (*P* < .0001; Fig 1, *C*), and CAT score (*P* = .005; Fig 1, *D*). Patients hospitalized with severe exacerbations also had higher sputum NET concentrations (*P* = .002). Very similar results were obtained with the DNA-elastase assay (see Fig E4 in this article's Online Repository at www.jacionline.org). The MPO-DNA assay had a limited dynamic range and was not considered further.

Soluble sputum NET concentrations were not correlated with age, smoking pack years, body mass index, or use of the antiplatelet agents aspirin or clopidogrel by using any assay. In multivariable analysis sputum NET concentrations were independently associated with percent predicted FEV_1_ in multiple linear regression (estimate, −0.19 per 100-unit change in NET concentration; *P* = .03). Histone-elastase levels were also independently associated with percent predicted FEV_1_ (*P* = .01). Sputum NET concentrations using both DNA-elastase and histone-elastase assays correlated with sputum neutrophil counts identified on cytospin preparations (*P* < .0001) and sputum EN-RAGE (*P* < .0001), cfDNA (*P* < .0001), MPO (*P* < .0001), and neutrophil elastase (*P* < .0001) levels. These results indicate that the abundance of NETs in sputum correlate with disease severity. NET concentrations in sputum were compared with the concurrent presence of NETs in peripheral blood to determine whether this was simply a reflection of systemic inflammation. On average, circulating DNA-elastase concentrations were 10,000-fold lower than in sputum and did not correlate with any markers of disease severity (data not shown).

To determine whether sputum NET concentration could be used as a predictive biomarker, we used receiver operating characteristic analysis. This showed that the optimal cutoff to identify frequently exacerbating patients (≥2 per year) was greater than 0.98 U/mL DNA-elastase complexes. Based on this cutoff, sputum NET concentration predicted time to next exacerbation (*P* < .0001) by using Kaplan-Meier survival analysis. Similarly, a cutoff of greater than 0.34 U/mL histone-elastase complexes predicted time to next exacerbation (*P* < .0001). In multivariable analysis DNA-elastase and histone-elastase complexes were associated with exacerbation frequency, even after adjustment for confounders (1.03 [95% CI, 1.01-1.06] per 0.1-unit increase [*P* = .02] and 1.04 [95% CI, 1.02-1.07] per 0.1-unit increase [*P* = .007], respectively). Included confounders were age, sex, smoking status, body mass index, FEV_1_ percent predicted, MRC Dyspnoea Score, and use of inhaled corticosteroids (ICSs).

### Other sputum markers of severity

**Fig 2:**
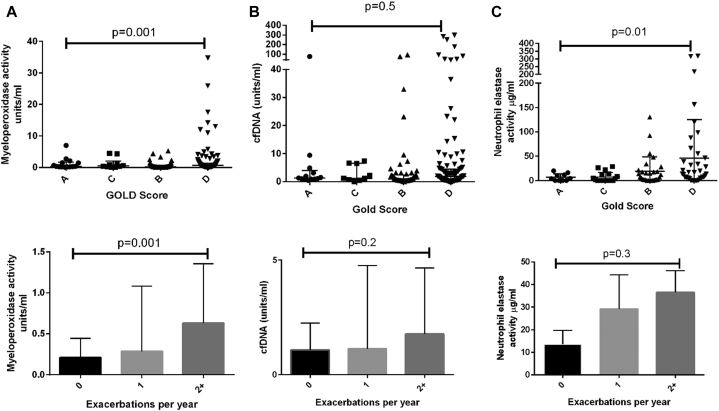
Sputum biomarkers and severity of COPD. **A,** Sputum MPO activity is associated with GOLD stage and exacerbation frequency (n = 99). **B,** Sputum cfDNA is not significantly associated with GOLD stage or exacerbations (n = 99). **C,** Neutrophil elastase activity is associated with GOLD stage but not significantly with exacerbation frequency (n = 99).

cfDNA was not associated with exacerbation frequency or GOLD score but was associated with sputum color (see Table E1 in this article's Online Repository at www.jacionline.org). Other NET markers, including elastase, MPO, and EN-RAGE, were associated with severity markers, including exacerbations, percent predicted FEV_1_, and GOLD score, but generally, the relationships were weaker for these nonspecific assays than for the NET assays (Fig 2 and see Table E1). Neutrophil elastase activity was associated with GOLD stage but not significantly with exacerbations.

### Sputum NET concentration is associated with microbiota composition

**Fig 3:**
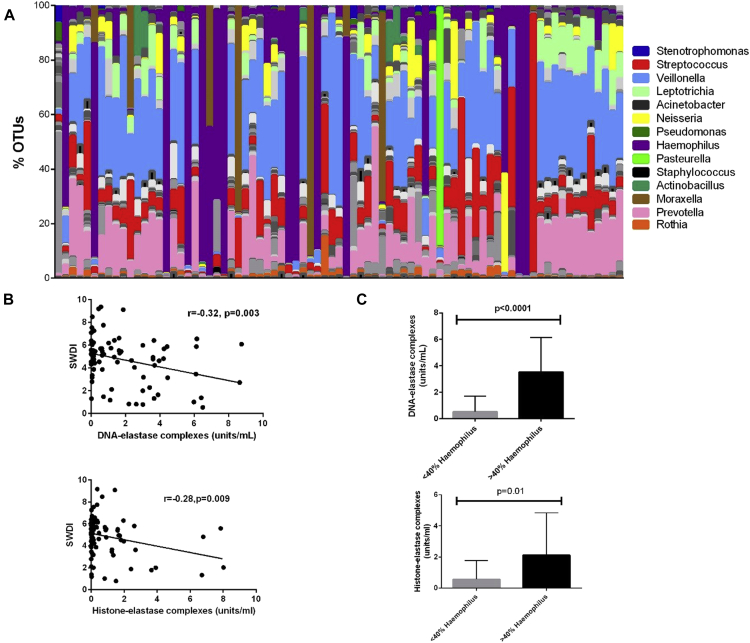
Microbiota composition and NET formation in patients with stable COPD. In the above figure, each *bar* represents an individual patient. **A,** Microbiomes of patients with COPD when clinically stable, with 14 of the most commonly identified genera per patient highlighted. Each patient is only represented once. **B,** Correlation of SWDI values of all stable samples against NET complexes (n = 89). **C,** NET formation in stable soluble sputum samples stratified by percentage of *Haemophilus* species OTUs present (n = 82).

**Fig 4:**
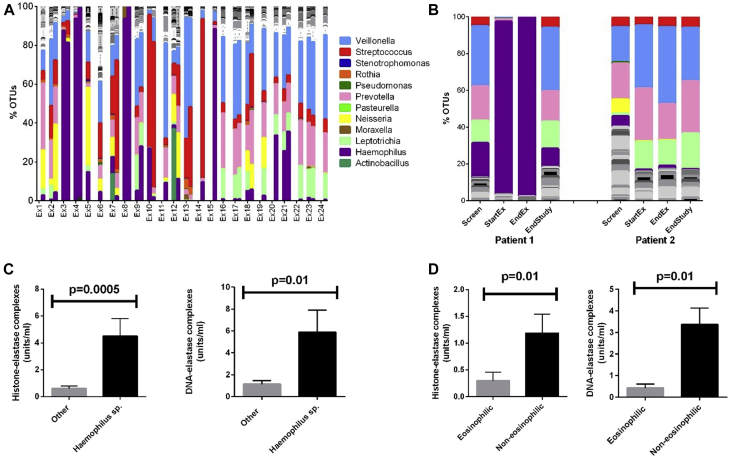
Changes in microbiota and NET formation at COPD exacerbation. **A,** Individual microbiome profiles of all exacerbation samples (n = 37 from 24 separate exacerbation events), with corresponding start and end exacerbation samples adjacent to each other. Exacerbation number denotes start of exacerbation sample, with end of exacerbation sample positioned to its right. Some patients were unable to produce sputum at both visits. **B,** Examples of longitudinal changes in microbiomes over time, showing 2 individual patients. *EndEx*, Ten days after exacerbation treatment once clinical recovery has occurred; *StartEx*, onset of exacerbation before treatment. **C,***Haemophilus* species OTU dominance at exacerbation is associated with significantly higher NET formation (n = 24). **D,** Based on blood eosinophilia,^26^ NET concentrations were increased in patients with noneosinophilic exacerbations and not in those with eosinophilic exacerbations (n = 24).

**Fig E5:**
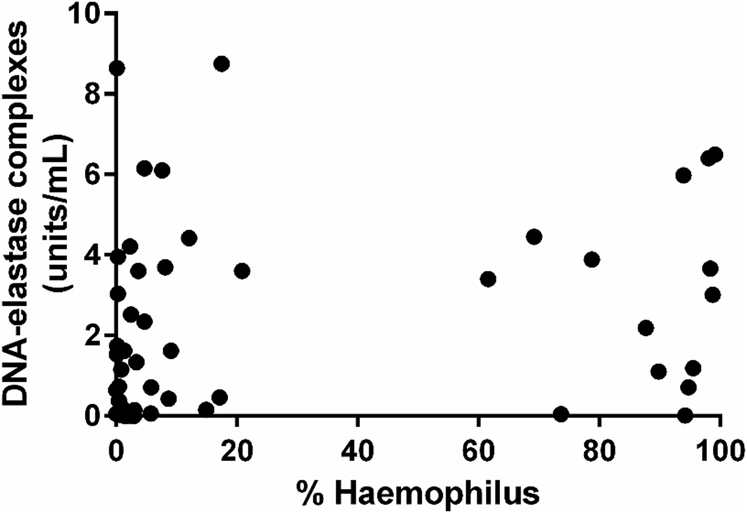


Results of 16S rRNA sequencing of DNA from whole sputum from patients with stable and exacerbating COPD is shown in Figs 3 and 4, respectively. The SWDI was used as a measure of the richness and evenness of the bacterial population found in sputum; a lower index indicates fewer species and more unevenness within a sample. Increasing sputum NET concentration was associated with a decreasing SWDI value in stable patients by using both DNA-elastase and histone-elastase assays (Fig 3). In patients with stable disease and also during exacerbations, *Haemophilus* species was most frequently the dominant pathogenic genus in patients with reduced species diversity. When stratified by the presence of greater than 40% *Haemophilus* species operational taxonomic units (OTUs) at the genus level (based on Fig E5 in this article's Online Repository at www.jacionline.org), there was a clear relationship between *Haemophilus* species dominance and NET formation, as measured based on histone-elastase complexes (*P* < .0001) and DNA-elastase complexes (*P* = .01; Fig 3, *C*).

**Fig E6:**
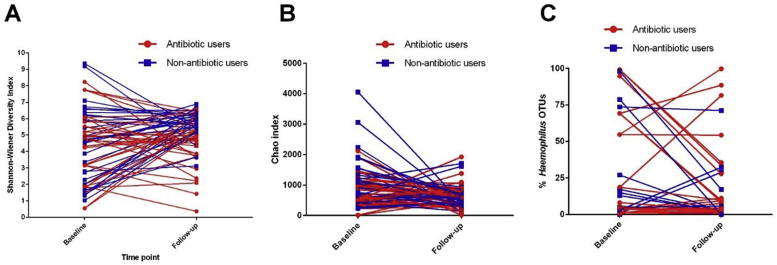


**Fig E7:**
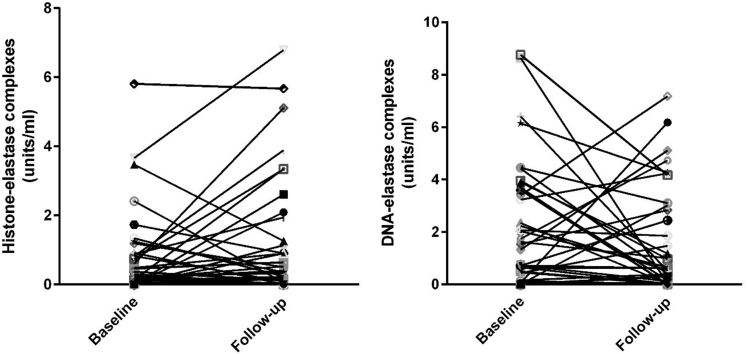


We investigated microbiota dynamics over the study period to determine whether antibiotic therapy might be responsible for reductions in SWDI values or *Haemophilus* species dysbiosis. Comparing patients who did and did not receive antibiotic therapy during 6 months of follow-up, there were no significant differences in change in SWDI, Chao index, or percentage of *Haemophilus* species OTUs (see Fig E6 in this article's Online Repository at www.jacionline.org). High variability in NET concentrations between baseline and follow-up was observed, but this was not statistically significant (see Fig E7 in this article's Online Repository at www.jacionline.org).

### NET concentrations during COPD exacerbations

Sixty-three exacerbations requiring antibiotic and corticosteroid treatment occurred during the study period in 39 patients. We studied a convenience sample of 24 exacerbations in which patients could be reviewed and sampled before administration of treatment. We quantified NETs in induced sputum at onset and after treatment of exacerbation (Fig 4, *A*). Exacerbations were heterogeneous, with microbiota profiling demonstrating that some exacerbations were associated with loss of bacterial diversity and others showing no change in overall microbiota profile (Fig 4, *B*). There was an association between DNA-elastase complexes and SWDI values during exacerbations (*R* = 0.48, *P* = .02). There was a significant association between sputum NET concentrations and exacerbation severity, as measured by using CAT (*R* = 0.35, *P* = .005) and St Georges respiratory questionnaire (*R* = 0.25, *P* = .01) score. Similar results were observed with the histone-elastase assay. NET concentrations were significantly increased in exacerbations where *Haemophilus* species was dominant (*P* = .01 for DNA-elastase and *P* = .0005 for histone-elastase; Fig 4, *C*). Classifying exacerbations as eosinophilic or noneosinophilic, as described by Bafadhel et al,^27^ there was a clear excess of NETs in sputum in patients with noneosinophilic exacerbations compared with low levels during eosinophilic exacerbations (*P* = .01 for both assays; Fig 4, *D*), which is consistent with the premise that the underlying pathologic disease process in these exacerbations might be different.

### Investigating potential mechanisms of NET formation in patients with COPD implicates failure of phagocytosis

**Fig 5:**
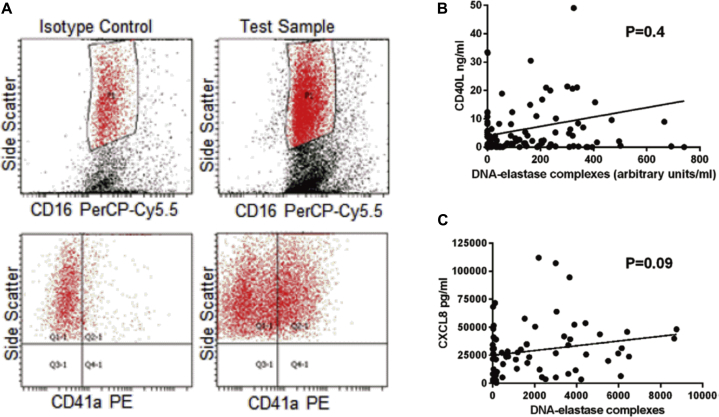
Platelet-neutrophil aggregates are present in airways of patients with COPD. **A,** Neutrophils were gated based on CD16 and side scatter *(top left)*, the quadrants for positive and negative CD41a were set *(bottom left)* in the isotype control, and these gates were applied to the test sample *(right panels)*. The example shows positive staining for the platelet marker CD41a-PE. **B,** No relationship between DNA-elastase complexes and soluble CD40 ligand, a marker of platelet activation (n = 72). **C,** No relationship between sputum NET concentrations and CXCL8 levels in sputum (n = 72). *PE*, Phycoerythrin; *PerCP*, peridinin-chlorophyll-protein complex.

We investigated a number of recognized NET triggers of relevance to COPD, such as CXCL8,^28^ complement component C5a, bacterial infection (described above), and activated platelets.^23^ We observed no relationship between sputum CXCL8 levels and NET concentrations (Fig 5, *C*). Complement component C5a was not detectable in the majority of sputum samples by using ELISA (data not shown). Markers of platelet activation were significantly increased in sera from patients with COPD (CD40 ligand [Fig 5, *B*] and P-selectin); analysis of sputum by means of flow cytometry showed the presence of neutrophil-platelet aggregates (Fig 5, *A*), but we found no evidence of a correlation between the degree of platelet activation and NET formation (correlation between NET concentrations and CD41a^+^ neutrophils, *P* = .053). A subanalysis in patients with and without treatment with antiplatelet drugs confirmed these findings. Similar results were observed with the histone-elastase assay (data not shown).

**Fig 6:**
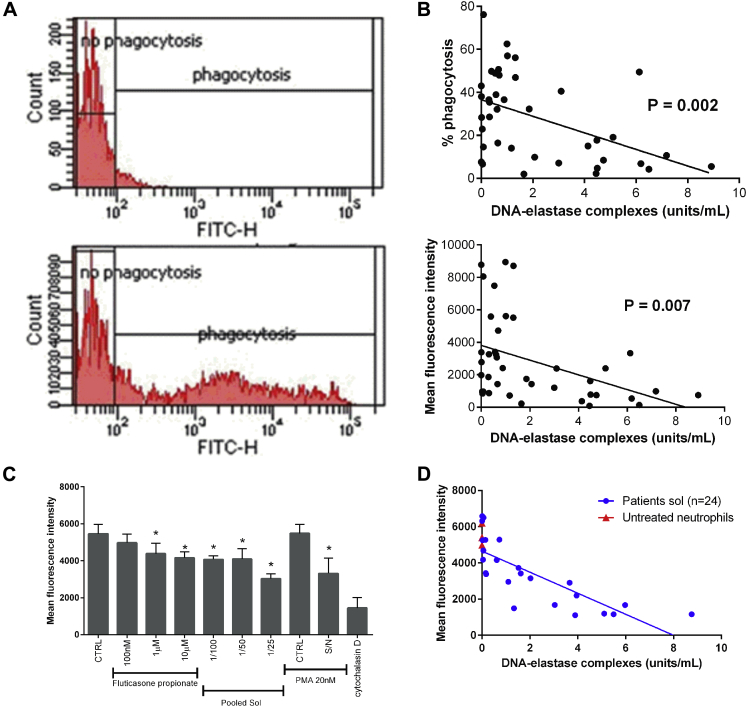
Direct relationship between sputum DNA-elastase complexes and *ex vivo* phagocytosis assessed by using flow cytometry. **A,** Representative image of phagocytosis flow cytometry showing isotype control *(top image)* used to set gates for test sample *(bottom image)*; test sample results were normalized by using isotype control to account for background fluorescence. **B,** Phagocytosis of fluorescein isothiocyanate–labeled *P aeruginosa* was evaluated by the percentage of positive cells and mean fluorescence, which quantifies the number of fluorescent bacteria ingested per cell (n = 40). **C,** Dose-dependent inhibition of phagocytosis of fluorescein isothiocyanate–labeled *E coli* by healthy donor neutrophils in response to pretreatment for 30 minutes with fluticasone propionate, pooled soluble sputum (n = 7 donors with COPD), or supernatant from neutrophils induced to undergo NETosis by means of incubation for 4 hours with 20 nmol/L PMA (n = 4 replicates with different donors for each experiment). *P < .05. **D,** Individual soluble sputum from 24 patients with COPD used to pretreat healthy donor neutrophils, followed by phagocytosis of fluorescein isothiocyanate–labeled *E coli* for 30 minutes. Data show a direct relationship between the sputum DNA-elastase complex concentration and subsequent neutrophil phagocytosis, suggesting that samples with high NET concentrations inhibit phagocytosis.

**Fig E8:**
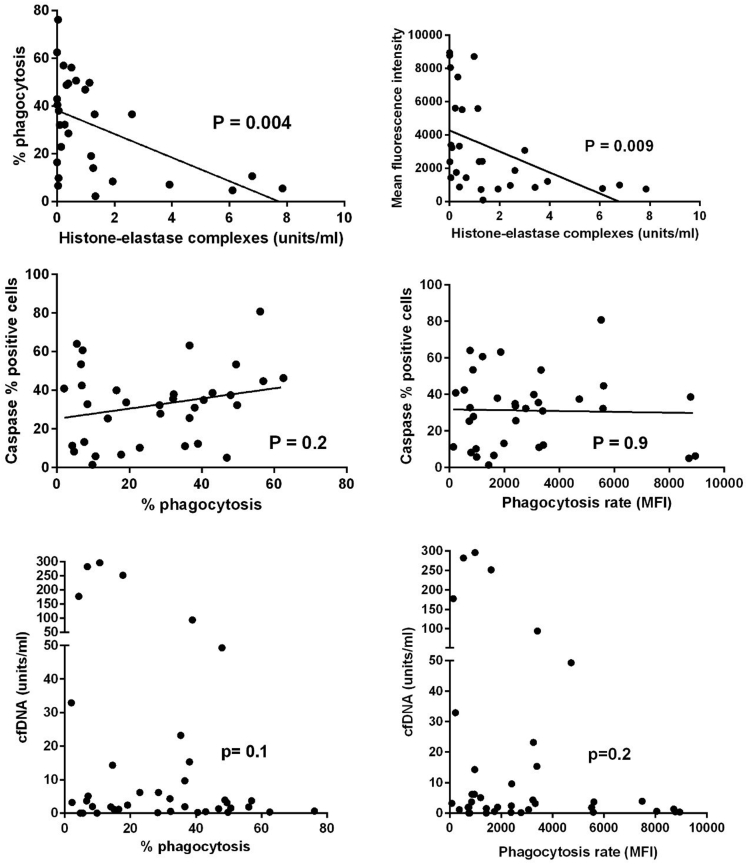


Branzk et al^29^ demonstrated that NET formation in bacterial infection only occurred when phagocytosis was inhibited. The phagocytic capacity of neutrophils, monocytes, and alveolar macrophages in patients with COPD has been extensively studied,^30^ and there is abundant evidence that phagocytosis is compromised in patients with this disease. Therefore we evaluated airway neutrophil phagocytosis of fluorescein isothiocyanate–labeled *Pseudomonas aeruginosa* to test the hypothesis that impaired phagocytosis in the presence of airway bacteria can contribute to NET formation. We observed a failure of neutrophil phagocytosis in patients with high sputum DNA-elastase complex concentrations (*P* = .002 and *P* = .007; Fig 6, *B*). Near-identical results were obtained with the histone-elastase assay (see Fig E8 in this article's Online Repository at www.jacionline.org). The relationships between cfDNA, free sputum CXCL8, IL-1β, TNF-α, and EN-RAGE levels and phagocytosis were not statistically significant.

**Fig E9:**
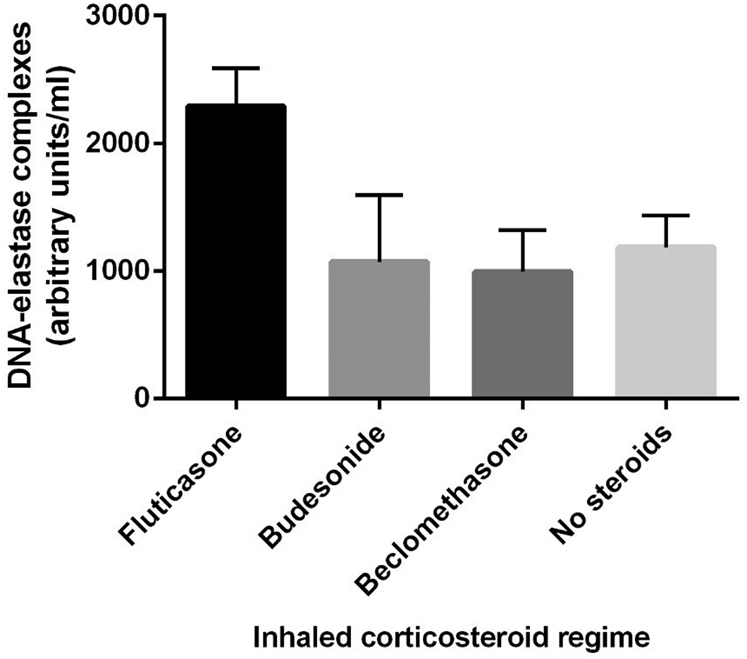


We excluded an effect of neutrophil viability on phagocytosis by demonstrating no correlation between caspase-positive cells and phagocytosis. We also observed a correlation between NET formation and daily beclomethasone dose equivalent of ICSs. The relationship was explained by a higher level of NET formation in patients receiving fluticasone propionate/salmeterol (*P* = .01, see Fig E9 in this article's Online Repository at www.jacionline.org). *In vitro* we found that fluticasone propionate at therapeutically relevant doses inhibited neutrophil phagocytosis of fluorescein isothiocyanate-labeled *Escherichia coli* (Fig 6, *C*) and that pooled sputum from patients with COPD similarly reduced phagocytosis (this pool was formed of patients not receiving ICSs to exclude the possibility of inhaled drug in the samples affecting neutrophil function).

To test the hypothesis that neutrophil products released into the sputum were responsible for phagocytosis inhibition, we treated blood neutrophils with PMA for 4 hours to induce NET formation and harvested the supernatant. This supernatant also demonstrated a dose-dependent inhibition of neutrophil phagocytosis after 30 minutes of incubation (a time point too early for NET formation in response to PMA to have occurred). The positive control, cytochalasin D, inhibited phagocytosis, as expected.

## Discussion

This study shows NET formation is present in patients with COPD and that NET concentrations are associated with disease severity in patients with COPD, with higher levels of NET complexes in patients with more severe disease when classified by the composite GOLD severity score, which incorporates percent predicted FEV_1_, symptoms, and the frequency of exacerbations. During exacerbations, increased NET concentrations were associated with noneosinophilic exacerbations and reduced bacterial diversity driven by increased *Haemophilus* species.

Therefore NETs appear to be potential biomarkers of disease severity and microbial dysbiosis in patients with COPD. Further work is required to address whether NETs directly contribute to disease progression in patients with COPD or are a reflection of more severe lung damage and associated alterations in the microbiota.

Neutrophil killing is critical to defense against bacterial and fungal pathogens in the lung. It is now known that neutrophils are able to alter their killing method, for example by sensing pathogen size and releasing NETs in response to large pathogens.^29^ The killing method appears to be binary because Branzk et al^29^ showed that NET formation was inhibited by phagocytosis through sequestration of neutrophil elastase, which is required to translocate to the nucleus to initiate NET formation.^29^, ^31^ *In vivo* evidence of this dichotomous neutrophil behavior has not previously been shown during human infections.

Microscopy studies have now demonstrated the presence of NETs in the airways of patients with cystic fibrosis, COPD, and asthma.^14^, ^15^, ^16^, ^32^, ^33^ Therefore the questions is not whether NETs are present but whether they are important in the progression of the disease. In addition, we must determining what drives NET formation in the airways of patients with COPD.

The number of recognized triggers for NET formation in *in vitro* systems is vast and includes proinflammatory cytokines (CXCL8 and TNF-α), bacterial products (formylated peptides and LPS), bacteria (*P aeruginosa* and *Haemophilus influenzae*), fungi, activated platelets, and rheumatoid factor (immunoglobulin).^11^, ^23^, ^29^, ^31^, ^33^, ^34^, ^35^, ^36^ Evaluating the drivers of NET formation in patients with COPD is challenging because the majority of these proposed drivers are present in the airways of patients with COPD under normal conditions.^5^ In this study NET concentrations were correlated most strongly during both stable COPD and exacerbations with the presence of *Haemophilus* species OTUs. Juneau et al^37^ previously demonstrated that *H influenzae* was able to induce NETs directly, and it has been shown that *H influenzae* might survive in NETs through production of nucleases and resistance to NET killing. Our study was not designed to answer whether the association between *Haemophilus* species and NET concentrations is due to natural selection because of *H influenzae*'s resistance to NET killing.

Branzk et al^29^ observed that NET formation in response to gram-negative bacteria did not occur under normal conditions, where neutrophil bacterial interaction results in phagocytosis and intracellular clearance. When phagocytosis was prevented through a physical barrier, NET formation resulted.^29^ Therefore we hypothesized that phagocytosis would be impaired in airway neutrophils from patients with COPD to explain the exaggerated NET formation in patients with COPD. Our data showed a direct relationship between airway neutrophil phagocytosis and NETs. Experimentally, exposure to soluble sputum from patients with COPD inhibited phagocytosis in neutrophils from healthy subjects and could be replicated by using supernatants from donor neutrophils from healthy subjects who had been induced to undergo NETosis by PMA. We speculate that neutrophil activation and NET formation in patients with COPD can cause the release of mediators that inhibit phagocytosis, creating an airway environment that promotes NET formation.^38^, ^39^ This can be exacerbated by ICSs, which are widely used in patients with COPD, because we demonstrated that fluticasone propionate *in vitro* and *in vivo* was associated with reduced neutrophil phagocytosis.

Although the question of whether NETs are able to kill bacteria is controversial, it is clear that NETs are a less effective means of bacterial killing compared with phagocytosis and are associated with greater collateral damage.^36^ We speculate that this could explain the loss of bacterial diversity and increased abundance of *Haemophilus* species OTUs in patients with COPD. Larger longitudinal studies are needed to determine whether NET formation identifies a specific endotype in patients with COPD, irrespective of whether NET status fluctuates over time and whether loss of phagocytic ability and subsequent NET formation precedes changes in the lung microbiota.

We acknowledge some potential limitations of this study. The majority of data are cross-sectional, and we are unable to assess whether the presence of NETs leads to more rapid disease progression, such as a long-term decrease in FEV_1_. We performed a large number of correlations in this study, increasing the possibility that some of the weaker correlations might be statistically significant by chance. For this reason and because of the relatively small sample sizes in some of our analyses, there is a need for independent replication of our findings. Sputum was selected for microbiota analysis because of the less invasive method of collection compared with bronchoalveolar lavage; although it is accepted that a protected brush bronchoscopy would be preferred for monitoring the lower airway microbiota, sputum is more practical in a routine clinical environment. The results of our study are similar to previously published data acquired by using various sample types from both lungs from patients with COPD and healthy subjects, as reviewed in Dickson et al.^40^ It is not feasible to perform and quantify microscopic images in a very large number of patients, and it is unlikely to be translated into a point-of-care clinical test, whereas NET ELISAs, such as those described here, are potentially applicable in clinical practice.

Simplified *in vitro* systems do not necessarily reflect the complex lung environment, and therefore although we can demonstrate inhibitory effects of lung fluids from patients with COPD and drugs, such as fluticasone propionate, on neutrophil functions, such as phagocytosis, we acknowledge that such assays are highly simplified. Nevertheless, we have identified a correlation between reduced phagocytosis *ex vivo* in airway neutrophils, which might be more reflective of their true *in vivo* function.

The majority of patients with COPD are treated with ICSs and bronchodilators.^38^ ICSs target eosinophilic inflammation and effectively reduce exacerbations in patients with eosinophilic COPD.^38^, ^41^ Patients who do not have eosinophilic COPD have neutrophilic airway inflammation, and to date, we have limited therapies capable of targeting neutrophilic inflammation.^5^ Drugs targeting NETs are in development; inhibition of NET formation has been shown to be beneficial in experimental models of diverse clinical diseases from psoriasis to lupus.^42^, ^43^ Our data suggest that NETs should be further evaluated as a therapeutic target in patients with COPD.

In conclusion, NETs are associated with disease severity and exacerbation frequency in this COPD cohort. NETs are associated with microbial dysbiosis, and further longitudinal studies are needed to determine whether modulation of NETs might affect airway microbial dysbiosis and clinical outcomes.

**Key messages**

• NETs have been observed in the lungs of patients with COPD; their significance in terms of clinical outcomes and their effect on bacterial clearance in the airway have not been established.
• We show that NETS in sputum are associated with loss of microbiota diversity and impaired *ex vivo* neutrophil phagocytosis, suggesting a possible role in disease progression.
• Consistent with this, measurement of NETs in sputum identifies patients with worse lung function, poorer quality of life, and a higher risk of future exacerbations.

## Supplementary data

Online Repository text
