# Supplementary material for: Neutrophil extracellular traps are associated with disease severity and microbiota diversity in patients with chronic obstructive pulmonary disease
Source: J Allergy Clin Immunol. 2018 Jan;141(1):117–27. doi: 10.1016/j.jaci.2017.04.022 (PMC5751731; doi:10.1016/j.jaci.2017.04.022)
Supplement: Online Repository text [file mmc1.docx]

**Neutrophil Extracellular Traps are associated with disease severity and microbiota diversity in Chronic Obstructive Pulmonary Disease**

Alison J Dicker^1^PhD, Megan L Crichton^1^MFM, Eleanor G Pumphrey^1^, Andrew J Cassidy^1^PhD, Guillermo Suarez-Cuartin^2^MD, Oriol Sibila^2^MD, Elizabeth Furrie^1^PhD, Christopher J Fong^1^, Wasyla Ibrahim^1^, Gill Brady^1^BSc, Gisli G Einarsson^3^PhD, J Stuart Elborn^3,4^MD, Stuart Schembri^1^MD, Sara E Marshall^45^PhD, Colin NA Palmer^1^PhD, James D Chalmers^1^PhD.

1. Scottish Centre for Respiratory Research, University of Dundee, Ninewells Hospital and Medical School, Dundee, Scotland.
2. Respiratory Department, Hospital de la Santa Creu i Sant Pau, Institut d´Invesitgacio Biomedica (IIB) Sant Pau, Barcelona, Spain.
3. Centre for Infection and Immunity, School of Medicine, Dentistry and Biomedical Sciences, Queen’s University Belfast, Northern Ireland.
4. National Heart and Lung Institute, Imperial College London
5. The Wellcome Trust, 215 Euston Road, London, WN1 2BB

**Corresponding Author:** Dr James D Chalmers, Division of Molecular and Clinical Medicine, University of Dundee, Dundee, DD1 9SY. E-mail: jchalmers@dundee.ac.uk, phone: 01382 383642

**Contribution**: Conception and design: JDC, SEM, SS and CNAP. All authors participated in data analysis and interpretation of the data. All authors were involved in writing and revising the article prior to submission.

**Funding:** This study was funded by the Chief Scientist Office, Scotland Grant number ETM/262. James D Chalmers acknowledges fellowship support from the Wellcome Trust. Sara Marshall is an employee of the Wellcome Trust. The funding agencies had no other role in the preparation, review, or approval of the manuscript.

**Conflicts of interest:** All authors declare no conflicts of interest in relation to the present study.

**Running title:** Neutrophil extracellular traps in COPD

**Keywords:** Neutrophils, phagocytosis, COPD, *Haemophilus*, exacerbations

**Methods**

**Inclusion and Exclusion Criteria**

Patients were included if >40 years; a FEV_1_/FVC ratio <70% and with a clinical diagnosis of COPD. Exclusion criteria included the inability to give informed consent; previous adverse reaction to nebulised hypertonic saline; asthma; bronchiectasis on HRCT scanning; cystic fibrosis; active mycobacterial disease; and immunosuppression. Patients receiving long term antibiotic therapy or maintenance oral corticosteroid therapy at screening were also excluded; additionally, patients needed to be clinically stable and free of antibiotic or corticosteroid therapy for 4 weeks prior to enrolment.

**Data Collection**

All relevant medical history (comorbidities, current medications, significant past conditions, operations and diagnostic procedures) was recorded at screening. Inhaled corticosteroid (ICS) dose was converted to Beclomethasone daily dose equivalent for analysis

**Systemic and Airway Inflammation**

Sputum IL-1β, IL-8 and TNFα, Serum CD40L and P-selectin were measured using commercially available kits (R&D Systems, Abingdon, UK) according to manufacturers’ instructions. Sputum was processed by ultracentrifugation to obtain soluble sputum as previously described (E1). Soluble sputum was stored at -80°C until analysis. All sputum ELISAs were validated by spike and recovery experiments (E2). EN-RAGE was measured as follows: Plates we coated with 1 in 2000 rabbit polyclonal antibody to EN-RAGE (Abcam Ab37657) and washed 3 times with PBS 0.05% tween 20. Plates were blocked with 1% BSA in PBS for 1 hour and then diluted sputum samples or recombinant EN-RAGE standards (diluted in 1% BSA PBS) were added and incubated for 2 hours at room temperature. Plates were incubated with 1ug/ml mouse monoclonal detection antibody (MAB10522, R+D systems) in 1% BSA PBS, detected with anti-mouse HRP (R+D systems) and developed with TMB substrate with values read at 450nm in a microplate reader. Neutrophil elastase was measured using a kinetic assay employing the substrate N-Succinyl-Ala-Ala-Ala-*p*-nitroanilide as previously described (Sigma-Aldrich) (E3). Cell free DNA was measured by diluting sputum samples 1 In 100 in PBS and adding SYTOX green at a final concentration of 6µM. Fluorescence was measured at 538nm emission and 450nm excitation and results were compared to a standard curve made from pure DNA of known concentration.

**Fluorescein isothiocyanate (FITC) labelling of bacteria**

*Pseudomonas aeruginosa* strain PA01 and *Escherichia coli* strain (ATCC 25922) were grown on *Pseudomonas* isolation agar and nutrient agar plates respectively. Freshly isolated colonies were inoculated into 10mls of Luria-Bertani broth at cultured overnight at 37^o^C with gentle shaking. Cultures were sub cultured 1 in 10 and grown for 3 hours to enter the logarithmic growth phase. Cultures (OD_600_ = 0.1) were labelled with FITC as described below: Bacterial suspensions were serially diluted and plated out on agar plates to determine colony counts. Overnight cultures of bacteria were heat inactivated 60°C for 1 hour before centrifugation at 3000 × g for 15 mins and OD brought to 1 with cold PBS. Samples were washed with 1% BSA-Hanks’ balanced salt solution (HBSS) containing Ca and Mg (Thermo Fisher). 100µL of FITC solution (0.5mg/ml in PBS) was added and the bacteria placed on a rotary mixer at 4^o^C for 30 mins. 900µl of ice cold 1%BSA-HBSS was added and samples were centrifuged at 10000rpm for 2 minutes, then re-suspended in 1mL 1%BSA-HBSS. Labelling was confirmed by flow cytometry and microscopy. Labelled bacteria were stored at -80°C until use.

**Phagocytosis assays**

Phagocytosis of peripheral blood and airway neutrophils was assessed using a standard flow cytometry based assay (E4): FITC labelled *Pseudomonas aeruginosa* (strain PA01) and *Escherichia coli* (ATCC 25922) were opsonised with 25% pooled healthy donor serum at 37^o^C for 1 hour. Opsonised bacteria were then added to patient neutrophils at a multiplicity of infection 10:1 to neutrophils (0.5 x 10^6^ per experiment). Phagocytosis was permitted at 37^o^C for up to 30 mins and then terminated by placing the samples on ice. Excess bacteria were removed by washing with PBS and cells subsequently analysed by flow cytometry. To differentiate phagocytosed (intracellular) bacteria from adherent (extracellular bacteria), cells were incubated with 0.1% trypan blue to quench extracellular fluorescence. A minimum of 10,000 events were counted. Results were expressed as the normalised rate of phagocytosis and mean fluorescent intensity for each sample. For some of these experiments, neutrophil phagocytosis was assessed after treatment with COPD patients’ soluble sputum (at a final concentration ranging from 10% soluble sputum to 0.01% in PBS), supernatant from phorbol 12-myristate 13-acetate (PMA) treated neutrophils or controls (PBS or Cytochalasin D) at 37^o^C.

For analysis of sputum neutrophils, the protocol was modified as follows; whole sputum was incubated at room temperature in PBS with 5% normal human serum, centrifuged at 20 x g for 10 mins then filtered through 48µM nylon gauze. 1 x 10^6 sputum neutrophils were incubated with FITC labelled PA01 bacteria or control for 30 mins at 37°C at a multiplicity of infection of 10:1 as described above, washed in PBS and analysed as above.

**Sputum neutrophil platelet aggregates**

Sputum neutrophil platelet aggregates were measured by flow cytometry. Sputum neutrophils were isolated from sputum as for the phagocytosis assay above, aggregated were visualised by co-staining the neutrophils with CD16 and CD41a (BD biosciences), gating of the neutrophils based on forward scatter, side scatter and CD16 then the normalised rate of platelet binding (CD41a) determined against an isotype control. A minimum of 10,000 events were counted.

**Sputum Neutrophil Cytospins**

Sputum neutrophils were isolated from sputum by filtering through a 48µM nylon gauze as described above, the concentration of cells in 100µL PBS adjusted to 30,000 cells per cytospin. The microscope slides were assembled with filter paper and cytofunnels then pre-wetted with 50µL PBS by centrifugation at 1000 rpm for 1 min in a cytocentrifuge (Shandon). Cells were added to cytofunnel and centrifuged for 1200 rpm for 3 mins. Slides were removed, allowed to air dry, stained with DiffQuik, dried, fixed and mounted before differential counts were determined.

**NET Assay with purified neutrophils**

NETs were studied using a fluorescent assay (E5) as follows: In 96 well plates, 5 x 10^4^ isolated blood neutrophils were added per well in HBSS containing Ca and Mg (ThermoFisher) and 20mM Hepes. After adhering for 30 mins, cells were treated with PMA at concentrations 1-100nM to induce NET formation. In some of these experiments, diphenyleneiodonium (DPI), a NADPH oxidase inhibitor that blocks NETosis, was added at 100nM for 30 mins prior to stimulation with PMA. After 4 hours at 37°C, NETs were stained with SYTOX green (10uM final concentration). Extracellular DNA was quantified by mean fluorescence and shown to correlate (r^2^ >0.95) with NET quantification by fluorescence microscopy (E5).

**Sputum microbiota sample preparation**

DNA and RNA was extracted from whole sputum using the AllPrep DNA/RNA Mini kit on the QIAcube automation platform (QIAGEN) as follows: Whole sputum was incubated in an equal volume of 1 in 10 diluted Sputolysin (Calbiochem) in a shaking incubator for 30mins at 37°C, mixed with Buffer RLT as per the AllPrep kit protocol, then passed through QIAshredder columns (QIAGEN) with the resulting supernatant undergoing sequential DNA and RNA extraction on the QIAcube. Quality and quantity of the DNA and RNA was determined by Nanodrop and Qubit machine, using the Qubit dsDNA broad range kit (Thermo Scientific). Metagenomic sequencing of the bacterial 16S rRNA gene was performed following the protocol in the Illumina library prep guide (https://www.illumina.com/content/dam/illumina-support/documents/documentation/chemistry_documentation/16s/16s-metagenomic-library-prep-guide-15044223-b.pdf), using primers targeting the V3 and V4 region (E6). Nextera XT Indices were added to each sample to allow multiplexing and the libraries sequenced using 2 x 300 paired end sequencing on the MiSeq platform using a MiSeq V3 kit (Illumina). Following sequencing on the Illumina MiSeq platform, FastQ files were imported into QIIME (version 1.9.0) and quality of reads checked; any reads with a Phred quality score less than Q20 were excluded when paired end reads were joined together for each sample. Un-joined reads were excluded from subsequent analysis. Sequences were clustered into operational taxonomic units (OTUs) based on 97% sequence similarity using the UCLUST algorithm (E7), aligned against the Greengenes Core reference alignment (Version 13.8) (E8) using PyNAST (Version 1.2.2) (E9). Taxonomy of the OTUs was assigned using the Ribosomal Database Project Classifier (Version 2.2) with the *de novo* OTU picking option (E10). OTUs were filtered to remove singletons and unassigned OTUs, or OTUs identified as Eukaryota, Human and Cyanobacteria. The dataset was normalised to the lowest number of OTUs and the Shannon-Wiener Species Diversity Index (SWDI) of the samples determined. All sequence data generated in this project can be found on the NCBI Sequence Read Archive, accession number SRP073159, a table of OTUs identified is shown in this supplement (Table E2).

**Validation of NET assays**

A series of controls were performed to ensure that the assays used in this study were measuring NETs. Citrullinated histones are regarded as one of the most specific markers of NET formation and their presence is frequently used as evidence that NET formation has occurred (E11). We used a semi-quantitative ELISA for citrullinated histone H3 (CITH3) (Cayman Chemical) to identify sputum samples with NETs present. Levels of DNA-elastase or histone-elastase complexes were compared between samples with detectable CITH3, defined as above the lower limit of detection of the ELISA, and those with undetectable CITH3 (at 1 in 10).

To exclude the possibility that sample preparation methods may affect NET formation we compared the detection of NETs in samples from patients prepared by ultracentrifugation at 50,000g for 90 minutes with samples diluted in 4xPBS followed by standard centrifugation, and also tested the correlation of samples obtained from paired sputum and BAL from the same patient taken on the same day. Agreement was determined by linear regression and by the Bland-Altman method.

Finally, we investigated possible passive interaction between DNA and elastase or between DNA and other components in sputum (E12). Fish sperm DNA (Sigma Aldrich) was mixed with purified neutrophil elastase (Sigma Aldrich) at 37^o^C for 1 hour. Neutrophil elastase was added at a concentration of 3ug/ml based on the mean concentration of total elastase present in 10 sputum samples (measured by ELISA, Total neutrophil elastase, Assaypro EE1001-1). Increasing concentrations of DNA were added at 4, 20 and 40ug/ml based on concentrations of DNA measured in sputum samples. Passive association was measured by DNA-elastase ELISA as described in the main manuscript text.

To evaluate passive association *in vivo*, soluble sputum contained 120ng/ml neutrophil elastase was incubated at 37^o^C for 1 hour with DNA at 4, 20 and 40ug/ml and the effect of increasing excess DNA on the presence of DNA-elastase complexes was measured by ELISA.

To validate the Histone-elastase assay, we performed a degradation experiment using DNAse. If histones and elastase are indirectly associated as part of DNA based traps, then the levels of histone-elastase complexes should be reduced by treatment of sputum samples with DNAse. Conversely, if histones and elastase were passively associating and directly bound, then DNase treatment should have no effect on sputum levels of histone-elastase complexes. Sputum containing 10ug/ml of DNA was incubated with 0 to 5 units of DNAse, where 1 unit is the amount of DNAse required to degrade 1ug of DNA.

**Statistical Analysis**

Statistical analysis of data was carried out using SPSS 21 and GraphPad Prism 6.07. Multivariable analysis was conducted using logistic regression for categorical outcomes with model fit evaluated with the Hosner-Lemeshow goodness of fit test. Multiple linear regression was used for continuous outcomes and negative binomial models for analysis of exacerbations. Pre-specified confounders were age, gender, smoking status, BMI, FEV_1_% predicted, MRC dyspnoea score and use of inhaled corticosteroids (ICS). Biomarker method agreement was evaluated by linear regression and Bland-Altman plots. Statistical significance was set at P<0.05.

**Results**

**Validation of NET ELISA**

DNA-elastase complexes accurately quantified NETs induced in healthy control neutrophils by treatment with PMA whilst release of DNA-elastase complexes was inhibited by diphenyleneiodonium (DPI), which prevents NET formation through NADPH oxidase. Importantly, neutrophils lysed with 0.1% Triton X100 released negligible quantities of DNA-elastase complexes (Figure E1A). There was a direct correlation between the DNA-elastase and SYTOX fluorescence in healthy control blood neutrophils treated with PMA (Figure E1B). In patient samples, there was a strong correlation between MPO-DNA and DNA-elastase ELISAs, and between the DNA-elastase and Histone-elastase ELISAs (Figure E1C, r=0.81, p<0.0001 and figure E1D r=0.66, p<0.0001 respectively). Samples containing citrullinated histones (N=30) contained more DNA-elastase complexes and histone-elastase complexes (p<0.0001 for all comparisons) compared to samples without detectable citrullinated histones by ELISA (N=30) (Figures S1E and S1F respectively).

(FIG E1)

We found no evidence that sample preparation affected the formation of NETs, with a strong linear correlation demonstrated between samples split for ultracentrifugation and PBS dilution methods, and between sputum and BAL samples taken from the same patient on the same day (Figure E2).

(FIG E2)

We found little evidence of non-specific interaction between DNA and elastase. Incubation of increasing concentrations of DNA with elastase resulted in only low levels of DNA-elastase complexes detectable by ELISA, at levels far below those detected in patients. Adding increasing concentrations of free DNA to sputum samples did not result in significant increases in DNA-elastase complexes over the levels present prior to addition of DNA (Figure E3A). As a specific control for the histone-elastase complex assay, we treated sputum with DNAse to determine if this could disrupt NET complexes. We demonstrated a dose dependent decrease in histone-elastase complexes with DNAse treatment (p=0.01) (Figure E3B).

(FIG E3)

**NETs are associated with clinical disease severity in stable COPD**

(FIG E4)

(TABLE E1)

**Sputum NET concentration is associated with microbiota composition**

(FIG E5)

**Impact of antibiotic therapy on diversity and Haemophilus dominance in COPD**

Sequential samples were studied in a subgroup of patients over the 6 month follow-up period. 28 patients had paired samples and received no antibiotic therapy between baseline and follow-up. 38 patients had paired samples and received at least one course of antibiotics between baseline and follow-up.

Changes between the two time-points in the Shannon-Wiener Species Diversity Index (A), Chao index (B) and % of *Haemophilus* OTU’s (C) are shown in Figure E6. During follow-up, mean change in SWDI was 0.93 for those not receiving antibiotics and 0.46 for those receiving antibiotics. Mean difference 0.47 95% CI -0.66 to 1.61, p=0.4. The proportion of patients experiencing a reduction in SWDI was also not significantly different between groups 36% for those not receiving antibiotics vs 50% for those receiving antibiotics, p=0.2.

(FIG E6)

In terms of the Chao index, mean change during follow-up was -416 for those not receiving antibiotics and -157 for those receiving antibiotics (mean difference -258 9% CI -635 to 117,p=0.2). The proportions showing a reduction in Chao index, were 67% vs 52%, p=0.2.

There was also no significance difference in Haemophilus OTUs between those treated with antibiotics and those not receiving antibiotics. Mean change was -8% for those receiving antibiotics and -12% for those not receiving antibiotics (mean difference -4% 9%CI -28 to 20, p=0.7).

We conclude from this analysis that antibiotic therapy does not have a large impact of diversity in the short term, but that our study was not powered to show small short term effects and was not designed to show longer term effects with repeated antibiotic courses over time.

In a second longitudinal analysis we compared DNA-elastase and histone-elastase complexes measured at baseline and follow-up visits to examine the stability of the marker when patients are clinically stable. We observed high variability as shown below in figure E7. There were, however, no significant differences between baseline and follow-up for either the histone-elastase (pairwise comparison p=0.6) and DNA elastase (p=0.2).

(FIG E7)

(FIG E8)

(TABLE E2)**Table E1**. Comparison of different methods of measuring NET components, NET complexes, and cytokines hypothesised as being important in lung inflammation and their association with clinical markers of COPD disease severity*p<0.05, **p<0.001, ***p<0.0001. In view of multiplicity of testing, all p-values should be interpreted with caution.

| Assay | Age | Exacerbations | MRC Dyspnoea Score | Long term Oxygen treatment | Sputum colour | % predicted FEV_1_ | CAT | SGRQ | GOLD score |
| --- | --- | --- | --- | --- | --- | --- | --- | --- | --- |
| **Assays specifically targeting NETS** |  |  |  |  |  |  |  |  |  |
| Histone-elastase NET | 0.13 | 0.27** | 0.20* | 0.28** | 0.31** | -0.31** | 0.23** | 0.27** | 0.30** |
| DNA-elastase NET | 0.05 | 0.28** | 0.27*** | 0.19* | 0.25** | -0.30** | 0.33*** | 0.37*** | 0.31*** |
| **Non-specific NET components** |  |  |  |  |  |  |  |  |  |
| cfDNA | 0.06 | 0.12 | 0.07 | 0.17* | 0.41*** | -0.15 | 0.06 | 0.14 | 0.14 |
| Elastase activity kinetic | 0.11 | 0.23* | 0.05 | 0.10 | 0.39*** | -0.20* | 0.15 | 0.19* | 0.21* |
| MPO activity | -0.03 | 0.21** | 0.02 | 0.17 | 0.43*** | -0.11 | 0.06 | 0.12 | 0.16* |
| EN-RAGE | 0.03 | 0.08 | 0.11 | 0.09 | 0.11 | -0.26** | 0.21** | 0.27*** | 0.23** |
| **Cytokines** |  |  |  |  |  |  |  |  |  |
| IL-1beta | 0.14* | 0.09 | 0.02 | 0.18* | 0.37*** | -0.14 | 0.10 | 0.14 | 0.19** |
| CXCL8 | 0.10 | 0.03 | 0.04 | 0.05 | 0.39*** | -0.14 | 0.14 | 0.16 | 0.20** |
| TNF-alpha | 0.10 | 0.15 | 0.07 | 0.28** | 0.40*** | -0.15 | 0.13 | 0.15 | 0.20** |
| **Cells** |  |  |  |  |  |  |  |  |  |
| Neutrophil cell count | 0.18 | 0.06 | 0.25* | 0.18 | 0.30** | -0.13 | 0.08 | 0.13 | 0.16 |

**Table E2**: List of OTUs identified in stable and exacerbating COPD sputum samples, classified at the genus level. If identification was not possible at genus level, the OTUs were classified at a higher taxonomic level. OTUs identified in less than 10 samples and with a maximum representation in a sample of 0.5% are excluded from this list.

| **Stable OTUs** | **Exacerbation OTUs** |
| --- | --- |
| *[Prevotella]* | *[Prevotella]* |
| *Acholeplasma* | *Achromobacter* |
| *Achromobacter* | *Acinetobacter* |
| *Acidocella* | *Actinobacillus* |
| *Acinetobacter* | *Actinomyces* |
| *Actinobacillus* | *Aggregatibacter* |
| *Actinomyces* | *Agrobacterium* |
| *Aeromocrobium* | *Anaerococcus* |
| *Aggregatibacter* | *Arsenicicoccus* |
| *Agrobacterium* | *Atopobium* |
| *Alloiococcus* | *Bacillus* |
| *Anaerococcus* | *Bacteroides* |
| *Anaerovorax* | *Bifidobacterium* |
| *Atopobium* | *Bulleidia* |
| *Bacillus* | *Burkholderia* |
| *Bacteroides* | *Butyrivibrio* |
| *Beijerinckia* | *Campylobacter* |
| *Bifidobacterium* | *Capnocytophaga* |
| *Bilophila* | *Cardiobacterium* |
| *Bradyrhizobium* | *Carnobacterium* |
| *Bulleidia* | *Catonella* |
| *Burkholderia* | *Chryseobacterium* |
| *Butyrivibrio* | *Corynebacterium* |
| *Campylobacter* | *Cryocola* |
| *Capnocytophaga* | *Curvibacter* |
| *Cardiobacterium* | *Delftia* |
| *Catonella* | *Dermacoccus* |
| *Chryseobacterium* | *Devosia* |
| *Chthonomonas* | *Dialister* |
| *Clostridium* | *Dokdonella* |
| *Comamonas* | *Eikenella* |
| *Corynebacterium* | *Elizabethkingia* |
| *Curvibacter* | *Enhydrobacter* |
| *Delftia* | *Enterococcus* |
| *Desulfobulbus* | *Ethanoligenens* |
| *Desulfovibrio* | *Filifactor* |
| *Devosia* | *Finegoldia* |
| *Dialister* | *Fusobacterium* |
| *Dokdonella* | *Gemella* |
| *Eikenella* | *Granulicatella* |
| *Elizabethkingia* | *Haemophilus* |
| *Enhydrobacter* | *Kingella* |
| *Erwinia* | *Klebsiella* |
| *Exiguobacterium* | *Kocuria* |
| *Filifactor* | *Lactobacillus* |
| *Finegoldia* | *Lactococcus* |
| *Flavisolibacter* | *Lautropia* |
| *Fluviicola* | *Leptotrichia* |
| *Fusobacterium* | *Megasphaera* |
| *Gemella* | *Methylobacterium* |
| *Gemmata* | *Microbacterium* |
| *Geobacillus* | *Micrococcus* |
| *Granulicatella* | *Mogibacterium* |
| *Haemophilus* | *Moraxella* |
| *Hymenobacter* | *Moryella* |
| *Jonquetella* | *Mycoplasma* |
| *Kaistobacter* | *Neisseria* |
| *Kingella* | *Nevskia* |
| *Lactobacillus* | *Novosphingobium* |
| *Lactococcus* | *Ochrobactrum* |
| *Lautropia* | *Oribacterium* |
| *Leptotrichia* | *Paludibacter* |
| *Lysinibacillus* | *Parachlamydia* |
| *Megasphaera* | *Paracoccus* |
| *Methylobacterium* | *Parvimonas* |
| *Microbacterium* | *Pasteurella* |
| *Mogibacterium* | *Pedobacter* |
| *Moraxella* | *Peptococcus* |
| *Moryella* | *Peptoniphilus* |
| *Mycobacterium* | *Peptostreptococcus* |
| *Mycoplasma* | *Phyllobacterium* |
| *Neisseria* | *Porphyromonas* |
| *Ochrobactrum* | *Prevotella* |
| *Oribacterium* | *Propionibacterium* |
| *Paenibacillus* | *Pseudomonas* |
| *Paludibacter* | *Pseudoramibacter_Eubacterium* |
| *Parachlamydia* | *Psychrobacter* |
| *Paracoccus* | *Ralstonia* |
| *Parvimonas* | *Rhodococcus* |
| *Pasteurella* | *Rothia* |
| *Pedobacter* | *Ruminococcus* |
| *Peptococcus* | *Schwartzia* |
| *Peptoniphilus* | *Selenomonas* |
| *Peptostreptococcus* | *Slackia* |
| *Phyllobacterium* | *Sneathia* |
| *Porphyromonas* | *Sphaerochaeta* |
| *Prevotella* | *Sphingobacterium* |
| *Propionibacterium* | *Sphingomonas* |
| *Propionivibrio* | *Staphylococcus* |
| *Proteus* | *Stenotrophomonas* |
| *Pseudomonas* | *Streptococcus* |
| *Pseudoramibacter_Eubacterium* | *Tannerella* |
| *Pyramidobacter* | *Thermus* |
| *Ralstonia* | *Treponema* |
| *Rheinheimera* | Unknown [*Mogibacteriaceae*] |
| *Rhodococcus* | Unknown [*Mogibacteriaceae*] |
| *Roseateles* | Unknown [*Paraprevotellaceae* |
| *Roseburia* | Unknown *Acetobacteraceae* |
| *Rothia* | Unknown *Actinomycetaceae* |
| *Scardovia* | Unknown *Aerococcaceae* |
| *Schwartzia* | Unknown *Aeromonadaceae* |
| *Segetibacter* | Unknown *Alphaproteobacteria* |
| *Selenomonas* | Unknown *Bacilli* |
| *Sharpea* | Unknown *Bacteria* CW040 |
| *Slackia* | Unknown *Bacteria* EW055 |
| *Sneathia* | Unknown *Bacteria* F16 |
| *Sphaerochaeta* | Unknown *Bacteria* Rs-0445 |
| *Sphingobacterium* | Unknown *Bacteria* SR1 |
| *Sphingomonas* | Unknown *Bacteria* TM7-3 |
| *Sphingopyxis* | Unknown *Bacteria* WPS-2 |
| *Spirosoma* | Unknown *Bacteroidales* |
| *Staphylococcus* | Unknown *Bacteroidales* S24-7 |
| *Stenotrophomonas* | Unknown *Bifidobacteriaceae* |
| *Streptococcus* | Unknown *Bradyrhizobiaceae* |
| *Sutterella* | Unknown *Cardiobacteriaceae* |
| *Tannerella* | Unknown *Caulobacteraceae* |
| *Treponema* | Unknown *Chitinophagaceae* |
| Unknown [*Chloracidobacteria*] DS-100 | Unknown *Clostridiales* |
| Unknown [*Chloracidobacteria*] Ellin6075 | Unknown *Clostridiales* |
| Unknown [*Chloracidobacteria*] RB41 | Unknown *Comamonadaceae* |
| Unknown [*Mogibacteriaceae*] | Unknown *Coriobacteriaceae* |
| Unknown [*Paraprevotellaceae*] | Unknown *Dethiosulfovibrionaceae* |
| Unknown [T*issierellaceae*] | Unknown *Enterobacteriaceae* |
| Unknown [*Weeksellaceae*] | Unknown *Flavobacteriaceae* |
| Unknown *Acetobacteraceae* | Unknown *Gemellaceae* |
| Unknown *Acidimicrobiales* C111 | Unknown *Gemellaceae* |
| Unknown *Acidobacteria* | Unknown *Intrasporangiaceae* |
| Unknown *Actinomycetaceae* | Unknown *Lachnospiraceae* |
| Unknown *Actinomycetales* | Unknown *Lachnospiraceae* |
| Unknown *Aerococcaceae* | Unknown *Lactobacillales* |
| Unknown *Alcaligenaceae* | Unknown *Moraxellaceae* |
| Unknown *Alphaproteobacteria* | Unknown *Neisseriaceae* |
| Unknown *Anaerolinaceae* SHD-231 | Unknown *Oxalobacteraceae* |
| Unknown *Aurantimonadaceae* | Unknown *Oxalobacteraceae* |
| Unknown *Bacilli* | Unknown *Pasteurellaceae* |
| Unknown *Bacteria* BD1-5 | Unknown *Peptococcaceae* |
| Unknown *Bacteria* CW040 | Unknown *Peptostreptococcacea* |
| Unknown *Bacteria* EW055 | Unknown *Phycisphaerales* |
| Unknown *Bacteria* F16 | Unknown *Propionibacteriaceae* |
| Unknown *Bacteria* Rs-045 | Unknown *Rickettsiales* |
| Unknown *Bacteria* SR1 | Unknown *Rickettsiales* |
| Unknown *Bacteria* TM7-3 | Unknown *Solirubrobacteraceae* |
| Unknown *Bacteroidales* | Unknown *Streptococcaceae* |
| Unknown *Bacteroidales* BE24 | Unknown *Veillonellaceae* |
| Unknown *Bacteroidales* S24-7 | Unknown *Vibrionaceae* |
| Unknown *Bifidobacteriaceae* | Unknown *Weeksellaceae* |
| Unknown *Caldilineaceae* | Unknown *Xanthomonadaceae* |
| Unknown *Campylobacterales* | Unknown *Xanthomonadaceae* |
| Unknown *Caulobacteraceae* | Unkown Bacteria BD1-5 |
| Unknown *Chitinophagaceae* | *Variovorax* |
| Unknown *Clostridiales* | *Veillonella* |
| Unknown *Clostridiales* |  |
| Unknown *Comamonadaceae* |  |
| Unknown *Coriabacteriaceae* |  |
| Unknown *Dethiosulfovibrionaceae* |  |
| Unknown *Enterobacteriaceae* |  |
| Unknown *Enterobacteriaceae* |  |
| Unknown *Firmicutes* |  |
| Unknown *Flavobacteriaceae* |  |
| Unknown *Gaiellaceae* |  |
| Unknown *Gaiellales* |  |
| Unknown *Gaiellales* AK1AB1_02E |  |
| Unknown *Gemellaceae* |  |
| Unknown *Geodermatophilaceae* |  |
| Unknown *Intrasporangiaceae* |  |
| Unknown *Koribacteraceae* |  |
| Unknown *Lachnocpiraceae* |  |
| Unknown *Lactobacillaceae* |  |
| Unknown *Lactobacillales* |  |
| Unknown *Leptotrichiaceae* |  |
| Unknown *Methylobacteriaceae* |  |
| Unknown *Microbacteriaceae* |  |
| Unknown *Microbacteriaceae* |  |
| Unknown *Micrococcaceae* |  |
| Unknown *Moraxellaceae* |  |
| Unknown *Myxococcales* OM27 |  |
| Unknown *Nacillaceae* |  |
| Unknown *Neisseriaceae* |  |
| Unknown *Neisseriaceae* |  |
| Unknown *Nocardioidaceae* |  |
| Unknown *Oxalobacteraceae* |  |
| Unknown *Oxalobacteraceae* |  |
| Unknown *Paenibacillaceae* |  |
| Unknown *Pasteurellaceae* |  |
| Unknown *Pasteurellaceae* |  |
| Unknown *Peptostreptococcaceae* |  |
| Unknown *Phycisphaerae* WD2101 |  |
| Unknown *Phyllobacteriaceae* |  |
| Unknown *Planococcaceae* |  |
| Unknown *Prevotellaceae* |  |
| Unknown *Propionibacteriaceae* |  |
| Unknown *Pseudomonadaceae* |  |
| Unknown *Rhizobiaceae* |  |
| Unknown *Rhodobiaceae* |  |
| Unknown *Rickettsiaceae* |  |
| Unknown *Rickettsiales* |  |
| Unknown *Rickettsiales* |  |
| Unknown *Rikenekkaceae* Blvii28 |  |
| Unknown *Ruminococcaceae* |  |
| Unknown *Sinobacteraceae* |  |
| Unknown *Solibacterales* |  |
| Unknown *Solirubrobacteraceae* |  |
| Unknown *Solirubrobacterales* |  |
| Unknown *Solirubrobacterales* |  |
| Unknown *Sphingomonadaceae* |  |
| Unknown *Sporichthyaceae* |  |
| Unknown *Tenericutes* ML615J-28 |  |
| Unknown *Thermomicrobia* JG30-KF-CM45 | |
| Unknown *Veillonellaceae* |  |
| Unknown *Veillonellaceae* |  |
| Unknown *Verrucomicrobiaceae* |  |
| Unknown *Xanthomonadaceae* |  |
| *Veillonella* |  |

**References**

E1. Chalmers JD, Smith MP, McHugh BJ, Doherty C, Govan JR, Hill AT. Short- and long-term antibiotic treatment reduces airway and systemic inflammation in non-cystic fibrosis bronchiectasis. *American Journal of Respiratory and Critical Care Medicine* 2012;186:657-665.

E2. Stockley RA, Bayley DL. Validation of assays for inflammatory mediators in sputum. *European Respiratory Journal* 2000;15:778-781.

E3. Chalmers JD, Smith MP, McHugh BJ, Doherty C, Govan JR, Hill AT. Short- and long-term antibiotic treatment reduces airway and systemic inflammation in non-cystic fibrosis bronchiectasis. *Am J Respir Crit Care Med* 2012;186:657-665.

E4. Farnworth SL, Henderson NC, Mackinnon AC, Atkinson KM, Wilkinson T, Dhaliwal K, et al. Galectin-3 reduces the severity of pneumococcal pneumonia by augmenting neutrophil function. *The American journal of pathology* 2008;172:395-405.

E5. Gray RD, Lucas CD, Mackellar A, Li F, Hiersemenzel K, Haslett C, et al. Activation of conventional protein kinase c (pkc) is critical in the generation of human neutrophil extracellular traps. *Journal of inflammation (London, England)* 2013;10:12.

E6. Klindworth A, Pruesse E, Schweer T, Peplies J, Quast C, Horn M, et al. Evaluation of general 16s ribosomal rna gene pcr primers for classical and next-generation sequencing-based diversity studies. *Nucleic Acids Research* 2013;41.

E7. Edgar RC. Search and clustering orders of magnitude faster than blast. *Bioinformatics (Oxford, England)* 2010;26:2460-2461.

E8. DeSantis TZ, Hugenholtz P, Larsen N, Rojas M, Brodie EL, Keller K, et al. Greengenes, a chimera-checked 16s rrna gene database and workbench compatible with arb. *Applied and environmental microbiology* 2006;72:5069-5072.

E9. Caporaso JG, Bittinger K, Bushman FD, DeSantis TZ, Andersen GL, Knight R. Pynast: A flexible tool for aligning sequences to a template alignment. *Bioinformatics (Oxford, England)* 2010;26:266-267.

E10. Wang Q, Garrity GM, Tiedje JM, Cole JR. Naive bayesian classifier for rapid assignment of rrna sequences into the new bacterial taxonomy. *Applied and environmental microbiology* 2007;73:5261-5267.

E11. Wong SL, Demers M, Martinod K, Gallant M, Wang Y, Goldfine AB, et al. Diabetes primes neutrophils to undergo netosis, which impairs wound healing. *Nat Med* 2015;21:815-819.

E12. Belorgey D, Bieth JG. DNA binds neutrophil elastase and mucus proteinase inhibitor and impairs their functional activity. *FEBS Lett* 1995;361:265-268.

**FIGURE LEGENDS**

**Figure E1**. Validation of an ELISA to measure neutrophil extracellular trap (NET) formation through detection of DNA-elastase and histone-elastase complexes. **A:** Quantification of NETs in neutrophils induced by phorbol 12-myristate 13-acetate and inhibited by diphenyleneiodonium (values shown are the mean and SEM of 3 independent experiments). **B:** Correlation of DNA-elastase complexes with SYTOX green quantification of extracellular DNA after neutrophils were induced to undergo NET formation with PMA, both of which are accepted proxies for NETs (n=7 samples). **C and D:** Correlation of DNA-elastase complexes with Histone-elastase complexes (n=162 samples) and MPO-DNA complexes (n=82 samples). **E and F:** citrullinated histone H3 positive samples contain DNA-elastase and Histone-elastase complexes, indicative of NET formation.

**Figure E2:** NETs are not induced due to sample preparation method. **A:** Correlation between different sputum samples methods. **B:** Bland Altman plots show acceptable agreement between sputum samples from the same patient prepared using ultracentrifugation and PBS dilution methods. **C:** Agreement between ultracentrifuged sputum and BAL. **D:** Bland Altman comparison between sputum and BAL in the same patient. Data are presented for the DNA-elastase assay. n=10 patient samples per comparison.

**Figure E3:** **A**: Limited evidence of passive interaction between DNA and elastase. **B:** DNAse treatment reduces the association between histone and elastase consistent with these being contained within NETs. Experiments shown are the mean (standard error of the mean) of 3 independent experiments.

**Figure E4**. DNA-elastase concentrations in soluble sputum of COPD patients are associated with clinical markers of COPD disease severity. **A:** NET concentration in stable samples compared to GOLD score (n=99). **B:** NET concentration compared to number of exacerbations reported by study patients in previous year (n=99). (SGRQ) (n=99). **C:** NET concentration in stable samples compared to percent predicted forced expiratory volume in 1 second (% predicted FEV_1_). **D:** NET concentration in stable samples compared to COPD assessment test (CAT) (n=99).

**Figure E5:** Two distinct clusters of sputum samples are apparent; the arbitrary cut-off of 40% *Haemophilus* spp. OTUs was chosen based on this data.

**Figure E6**: Impact of antibiotic therapy during the study on diversity **A:** Shannon Wiener Diversity Index. **B:** Chao evenness index. **C:** % *Haemophilus* spp OTUs.

**Figure E7**. Changes over time in levels of NET complexes.

**Figure E8**. Relationship between neutrophil assays and phagocytosis. The top 2 panels show Histone-elastase complexes, the middle panels caspase positive cells on flow cytometry, and the bottom panels cfDNA concentrations in sputum.

**Figure E9**. Relationship between inhaled corticosteroid regime and NET formation (P=0.01 by Kruskal Wallis test). 45% of study participants were receiving fluticasone, 6% budesonide containing regimes and 13% beclomethasone contained regimes.
